# Integrative multi-omics and Mendelian randomization analysis reveal SPP1^+^ tumor-associated macrophage-driven prognostic signature for hepatocellular carcinoma

**DOI:** 10.3389/fmolb.2025.1594610

**Published:** 2025-05-01

**Authors:** Kai Lei, Yichun Lei, Zeyao Wang, Zhixin Ye, Jiawei Liu, Wenhao Chen, Caihong Zhou, Jinmei Tan, Shuxian Chen, Yifan Zhang, Jiehui Tan

**Affiliations:** ^1^ Center of Hepato-Pancreato-Biliary Surgery, The First Affiliated Hospital, Sun Yat-sen University, Guangzhou, Guangdong, China; ^2^ School of Nursing, Jiangxi University of Chinese Medicine, Nanchang, Jiangxi, China; ^3^ Department of General Surgery, Hui Ya Hospital of The First Affiliated Hospital, Sun Yat-sen University, Huizhou, Guangdong, China; ^4^ Department of Obstetrics and Gynecology, The First Affiliated Hospital, Sun Yat-sen University, Guangzhou, Guangdong, China; ^5^ Department of Hepatobiliary Surgery, The Third Affiliated Hospital, Sun Yat-sen University, Guangzhou, Guangdong, China; ^6^ Division of Hepatobiliopancreatic Surgery, Department of General Surgery, Nanfang Hospital, Southern Medical University, Guangzhou, Guangdong, China; ^7^ Department of Intensive Care Unit, Wuchuan People’s Hospital, Zhanjiang, Guangdong, China

**Keywords:** hepatocellular carcinoma, SPP1^+^ TAMs, Mendelian randomization, prognostic signature, UBE2I

## Abstract

**Background:**

The SPP1^+^ tumor-associated macrophages (TAMs) have been implicated in tumor metastasis and immune evasion. However, the prognostic significance of SPP1^+^ TAMs in hepatocellular carcinoma (HCC) remains largely unexplored. This study aimed to identify SPP1^+^ TAMs-related genes and construct a model to predict overall survival (OS) in HCC patients.

**Methods:**

Single-cell RNA sequencing (scRNA-seq) datasets from HCC patients were analyzed to identify SPP1^+^ TAMs. SPP1^+^ TAMs-related risk score (STRS) was developed using Mendelian randomization (MR) analysis and Least Absolute Shrinkage and Selection Operator (LASSO) regression. HCC patients from the Cancer Genome Atlas (TCGA) and Gene Expression Omnibus (GEO) cohorts were stratified into high- and low-STRS groups based on STRS. Kaplan-Meier survival analysis, receiver operating characteristic (ROC) curve analysis, and functional enrichment analysis were performed to assess the prognostic value of STRS.

**Results:**

SPP1^+^ TAMs exhibited strong associations with immunosuppressive functions. 16 SPP1^+^ TAMs-related genes were used to construct STRS. Patients in the high-STRS group had significantly worse OS than those in the low-STRS group (*p* < 0.001). ROC analysis demonstrated robust predictive power, with AUC values ranging from 0.685 to 0.748 for 1-year OS, 0.717 to 0.739 for 2-year OS, and 0.719 to 0.738 for 3-year OS. The STRS model also exhibited strong predictive capability for the distinction of drug resistance.

**Conclusion:**

This study identified SPP1^+^ TAMs-related genes as key prognostic indicators in HCC. The STRS model provides an effective tool for predicting patient survival and may facilitate personalized treatment strategies for HCC. These findings enhance the understanding of TAMs-driven immune modulation in HCC and highlight potential therapeutic targets for improving patient outcomes.

## Introduction

Hepatocellular carcinoma (HCC), originating from liver cells, is the most common form of primary liver cancer and represents a significant global health challenge (Gomaa et al., 2008). This disease can be triggered by several risk factors, including HBV/HCV infections, non-alcoholic steatohepatitis (NASH), alcohol abuse, and smoking (Villanueva, 2019; Bray et al., 2018). Treatment options for HCC include surgery, transarterial chemoembolization, and radiation therapy, which can significantly improve patient survival rates (Vivarelli et al., 2013). Early-stage liver cancer can be treated with tumor resection and liver transplantation; however, many patients are not diagnosed until the disease has reached an advanced stage. Due to its high recurrence and metastasis rates, the five-year survival rate for liver cancer patients remains low. Given the high heterogeneity of HCC, predictive, preventive, and personalized medicine strategies are crucial for improving treatment outcomes. Therefore, it is imperative to uncover the mechanisms driving liver cancer progression and to identify effective biomarkers for personalized treatment of HCC patients.

Crosstalk between tumor cells and cells within the tumor microenvironment plays a critical role in tumor progression and influences therapeutic responses (Dunn et al., 2002). Among these, various populations of bone marrow-derived cells and lymphocytes are key players in inflammation, immune evasion, and responses to immunotherapy (Hackl et al., 2016; Ringelhan et al., 2018; Zhang et al., 2019). Under normal physiological conditions, hematopoietic stem cells in the bone marrow differentiate into various mature immune cell subsets, including macrophages, dendritic cells, and granulocytes. During the early stages of tumorigenesis, bone marrow cells effectively eliminate tumor cells through immune surveillance mechanisms (Condamine et al., 2015). However, as the tumor progresses, secreted growth factors reshape the differentiation process of bone marrow cells, inducing the generation of immunosuppressive regulatory cell subsets that further impair the host’s anti-tumor immune response (Hicks et al., 2022; Lasser et al., 2024). Single-cell RNA sequencing (scRNA-seq) has emerged as a powerful method for investigating the tumor microenvironment, particularly the immune landscapes of various cancers (Krishna et al., 2021; Sade-Feldman et al., 2018). Among these, SPP1^+^ tumor-associated macrophages (TAMs) have been frequently reported. For instance, Liu et al. identified SPP1^+^ TAMs as a key subset that promotes liver metastasis in colorectal cancer, influencing patient prognosis (Liu et al., 2022). In non-small cell lung cancer, SPP1^+^ TAMs have also been shown to correlate with poor prognosis (Leader et al., 2021). Although prognostic models related to macrophages have been established (Qu et al., 2022), models specifically associated with SPP1^+^ TAMs marker genes remain underexplored.

In this study, we identified SPP1^+^ TAMs through scRNA-seq data, performed Mendelian randomization (MR) analysis to determine related genes, and constructed the SPP1^+^ TAMs-related risk signature (STRS). We assessed the value of STRS in predicting prognosis for HCC patients and its potential to inform responses to immunotherapy. The findings of this study will provide deeper insights into the role of SPP1^+^ TAMs in HCC and contribute to the enhancement of personalized treatment strategies for HCC patients.

## Materials and methods

### Data collection

Transcriptomic data from the TCGA cohort were retrieved from the University of California Santa Cruz (UCSC) Xena data portal (https://xenabrowser.net) (Goldman et al., 2020). After removing duplicate samples, those lacking clinical information, and samples with a survival time of zero, a total of 363 HCC samples were included in the analysis. Additionally, microarray data and clinical characteristics from the GSE14520 dataset were obtained from the Gene Expression Omnibus (GEO) database (https://www.ncbi.nlm.nih.gov/geo/). ScRNA-seq data for HCC patients were sourced from GSE151530, GSE125449, and GSE149614.

### Developmental trajectory inference

To investigate functional heterogeneity and potential lineage differentiation within endothelial cell subsets, we employed the “Monocle3” R package (version 1.3.4) to reconstruct cellular trajectories (Qiu et al., 2017; Cao et al., 2019). The preprocessed single-cell expression matrix was converted into a “cell_data_set” object using the “new_cell_data_set” function in Monocle3. Highly variable genes were selected based on graph-based clustering results, which guided the construction of the trajectories. Dimensionality reduction was performed using the “learn_graph” function, which utilizes Principal Component Analysis (PCA), Uniform Manifold Approximation and Projection (UMAP), and t-distributed Stochastic Neighbor Embedding (t-SNE) for embedding. To infer the developmental trajectory, the root node was manually defined based on known biological markers or cluster characteristics, and cells were ordered along the pseudotime axis using the “order_cells” function. The resulting pseudotime trajectory provided valuable insights into the differentiation and functional diversity of endothelial cell subsets.

In addition to Monocle3, we used the “Slingshot” R package (version 2.7.0) to infer the developmental trajectories of endothelial cell subsets (Street et al., 2018). The data, reduced in dimensionality through UMAP via the Seurat package, was utilized for trajectory analysis. Cluster labels derived from Seurat’s clustering results were employed as inputs for the analysis. The Slingshot algorithm was then applied within a minimal spanning tree framework to connect clusters, followed by the fitting of smooth curves to model lineage trajectories. The “slingshot” function assigned pseudotime values to each cell along the inferred developmental lineages, with the root cluster determined by the trajectory structure and the biological relevance of the endothelial cell subsets. This approach allowed for the identification of potential lineage relationships and differentiation pathways within the endothelial cell subsets.

### Gene set testing

We employed the AUCell package (version 1.24.0) to calculate the AUCell scores for macrophage-related gene signatures in each individual cell (Aibar et al., 2017). We then calculated the scores for the following functional gene sets: Angiogenesis (Wu et al., 2022), Antigen Processing and Presentation (Kanehisa et al., 2025), M1 polarization (Sun et al., 2021), M2 polarization (Sun et al., 2021), and Phagocytosis (Wu et al., 2022).

### MR analysis

To elucidate the causal relationship between SPP1^+^ TAMs-related gene expression and HCC progression, we implemented a two-sample MR framework utilizing expression quantitative trait loci (eQTL) as instrumental variables (IVs). The eQTLs data were sourced from eQTLGen consortium database (https://eqtlgen.org/), which contained from 31,684 individuals (Vosa et al., 2021). Genetic instruments were systematically selected through cis-eQTL variants located within ±1 Mb of each gene’s transcription start site (Vosa et al., 2021). Corresponding genome-wide association study (GWAS) summary statistics for HCC were derived from the FinnGen Project (https://r10.risteys.finngen.fi/), comprising 500 cases and 314,193 control individuals (controls excluding all cancers).

SNPs significantly associated with gene expression (*p* < 0.05) were clumped (r^2^ < 0.01, 10,000-kb window) to ensure independence. Causal effects of gene expression on the progression of HCC were estimated using inverse-variance weighted (IVW) regression as the primary method, supplemented by MR-Egger regression, weighted median estimator (WME), simple mode, and weighted mode (Burgess et al., 2017; Long et al., 2023). Horizontal pleiotropy was assessed via MR-Egger intercept tests (*p* > 0.05 considered negligible), and heterogeneity across SNPs was quantified using Cochran’s Q statistic. Outlier SNPs identified by MR pleiotropy residual sum and outlier (MR-PRESSO) test (*p* < 0.05 for global test) were iteratively removed (Burgess et al., 2017).

### Construction of the STRS

Initially, genes associated with SPP1^+^TAMs were selected through MR analysis. The filtering criteria were as follows: a β value greater than 0, a p-value less than 0.05, a heterogeneity *p*-value (Heter.*p*) greater than 0.05, and an Egger intercept *p*-value greater than 0.05. After applying these filters, 31 genes remained for further analysis. To construct a more accurate prediction model, the Least Absolute Shrinkage and Selection Operator (LASSO) method was applied using the “glmnet” package (Friedman et al., 2010). This step generated a more precise model for predicting STRS, employing a Cox proportional hazards model for survival analysis. The STRS for each patient was then calculated using the following formula:

$$STRS=\sum_{i=1}^{n}Coef\left(\beta i\right)*\mathrm{Exp}\left(Xi\right)$$

In this formula, Coef (βi) represents the risk coefficient derived from the LASSO regression model, Exp (Xi) is the expression level of each gene selected by LASSO, and n is the total number of genes included in the final model. This formula sums the weighted gene expression values (where weights are the regression coefficients) to generate a continuous risk score for each patient. Based on the median STRS, HCC patients were categorized into low- and high-STRS groups. Kaplan-Meier survival analysis was performed using the “survival” (https://github.com/therneau/survival) and “survminer” (https://github.com/kassambara/survminer) R packages to assess the correlation between overall survival (OS) and STRS. Additionally, Receiver Operating Characteristic (ROC) curves were generated to evaluate the prognostic efficacy of the STRS.

### Drug sensitive analysis

The half-maximal inhibitory concentrations (IC50) of common antitumor drugs were predicted for both STRS groups using data from the Genomics of Drug Sensitivity in Cancer (GDSC) database (Yang et al., 2013). IC50 differences between the two groups were analyzed using the oncopredict R package (Maeser et al., 2021).

### Cell lines and cell culture

Human HCC Huh-7 cells were sourced from the China Center for Type Culture Collection (Shanghai, China). These cells were cultured in Dulbecco’s Modified Eagle’s Medium (DMEM, Gibco) supplemented with 10% fetal bovine serum (GIBCO, United States) and 1% penicillin-streptomycin (GIBCO, United States). Cultivation was performed in a 5% CO2 atmosphere at 37°C using an incubator (Thermo Scientific, United States).

### Knockdown of UBE2I in HCC cells

The pLKO.1 lentiviral vectors expressing short hairpin RNA (shRNA) targeting UBE2I were acquired from Umine Biotechnology Co., LTD (China). These shRNA constructs, along with the packaging plasmid (pCMV-ΔR8.9) and the envelope plasmid (pCMV-VSVG), were co-transfected into HEK293T cells using Lipofectamine 3000 (Invitrogen, United States). After 48 h of incubation, the viral supernatants were collected and used to infect Huh-7 cells in the presence of polybrene (8 μg/mL) (Solarbio, China). Stable cell lines were then selected by treatment with puromycin (2.5 μg/mL) for 48 h. The shRNA sequence utilized in this study is as follows: shUBE2I: TAAATTCGAACCACCATTATT.

### Western blot

After homogenization and centrifugation, the supernatant was collected for total protein quantification using the BCA protein assay kit (Solarbio, China). Fifty micrograms of protein from each sample were subjected to sodium dodecyl sulfate-polyacrylamide gel electrophoresis (SDS-PAGE) and subsequently transferred to polyvinylidene difluoride (PVDF) membranes. To block nonspecific binding, the membranes were incubated with 5% bovine serum albumin (BSA) prior to antibody incubation. The membranes were then incubated overnight at 4°C with primary antibodies (Proteintech, China). Afterward, the membranes were washed with TBS and incubated with horseradish peroxidase (HRP)-conjugated secondary antibodies in blocking buffer for 1 h at room temperature. Following three washes, protein bands were visualized using an enhanced chemiluminescence detection kit (Solarbio, China).

### Cell viability, migration and invasion assays

For the viability assay, 2000 cells per well were seeded in 96-well plates with fresh medium. Cell viability was assessed at 6, 24, 48, 72, 96, and 120 h using the Cell Counting Kit-8 (Dojindo, Japan), following the manufacturer’s instructions.

For the migration assay, 5 × 10^4 cells suspended in 500 μL of serum-free medium were placed in the upper chamber of transwell inserts (Corning Falcon, United States). The inserts were then placed in wells containing fresh medium supplemented with 20% fetal bovine serum to induce cell migration. After 48 h, the cells that had migrated to the lower surface were stained with 0.5% crystal violet and counted to evaluate cell migration.

Additionally, an invasion assay was performed similar to the migration assay. For this, a mixture of matri-gel was first added to the upper chamber of the transwell inserts. Following this, 5 × 10^4 cells suspended in 500 μL of serum-free fresh medium were added to the upper chamber. After 72 h, the invaded cells on the lower surface were stained with 0.5% crystal violet, and the number of invaded cells was counted to analyze cell invasion.

### Determination of apoptosis

Huh7 cells were plated in six-well plates, and apoptosis was assessed using an apoptosis detection kit according to the manufacturer’s instructions (Dojindo, Japan). The cells were harvested from the plates, and subpopulations were analyzed by flow cytometry. Initial gating was performed based on forward scatter (FSC) versus side scatter (SSC) to identify viable cells. Doublets were excluded using consecutive gating on FSC-Area versus FSC-Width and SSC-Area versus SSC-Width plots. Quadrant gates were set on the Annexin V/PI dot plot to classify the cells into four populations: living cells (Annexin V−/PI−), early apoptotic cells (Annexin V^+^/PI^−^), late apoptotic cells (Annexin V^+^/PI^+^), and necrotic cells (Annexin V^−^/PI^+^). The percentages of each cell population (Annexin V^+^/PI^−^, Annexin V^+^/PI^+^, Annexin V^−^/PI^+^, and Annexin V^−^/PI^−^) were determined based on the quadrant gates. Data analysis was performed using FlowJo v9.6.3 (TreeStar, Inc.).

### Statistical analysis

Statistical analyses were conducted using R (version 4.1.2). The Chi-square test or Fisher’s exact test was used for comparing categorical variables, while the Student’s t-test or Wilcoxon rank-sum test was applied to continuous variables. A *p*-value of less than 0.05 was considered statistically significant.

## Results

### Identification and classification of cell types in the HCC via single-cell analysis

**FIGURE 1:**
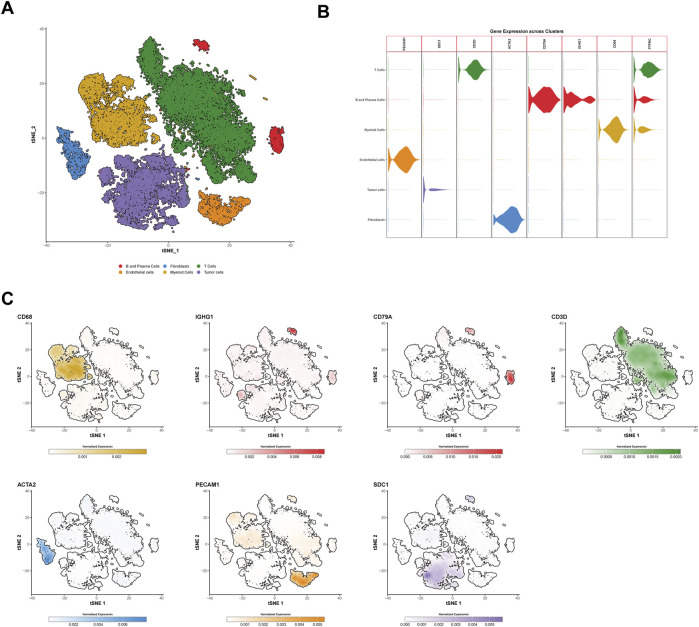
Identification and characterization of cell types in hepatocellular carcinoma (HCC) via single-cell RNA sequencing. **(A)** t-SNE clustering of 71,799 high-quality single cells from three public HCC datasets (GSE151530, GSE125449, and GSE149614). Cells are categorized into six major types: T cells (forestgreen), B and plasma cells (firebrick2), myeloid cells (goldenrod), endothelial cells (darkorange), fibroblasts (dodgerblue), and tumor cells (mediumpurple). **(B)** Violin plots showing the normalized gene expression of marker genes in different cell types. PECAM1 and ACTA2 are specific markers for endothelial cells and fibroblasts, respectively, while SDC1 is a tumor cell marker, and PTPRC is a general immune cell marker. Other markers such as CD3D, CD79A, and IGHG1 distinguish T/NK cells, B cells, and plasma cells, respectively. **(C)** t-SNE plots showing the expression of specific genes across different cell types. The color scale represents normalized gene expression levels, with darker colors indicating higher expression levels. The color scheme used in this figure is consistent with **(A,B)**. t-SNE, t-distributed Stochastic Neighbor Embedding.

To investigate the tumor microenvironment in HCC patients, we analyzed three publicly available single-cell transcriptome datasets (GSE151530, GSE125449, and GSE149614). After quality control and filtering, a total of 71,799 high-quality cells were obtained, with an average of 2,564 cells per sample and a mean of 25,714 genes detected per cell. Using the t-SNE method, the cells were classified into six major cell types: immune cells (T/NK cells, myeloid cells, and B/plasma cells), tumor cells, and stromal cells (endothelial cells and fibroblasts) (Figure 1A).

Tumor cells were identified using SDC1 as a marker, while immune cells were characterized using PTPRC. T/NK cells were distinguished by the marker CD3D, B cells were identified using CD79A, and plasma cells were labeled with IGHG1. Myeloid cells were defined by the expression of CD68. Stromal cells were categorized into endothelial cells and fibroblasts using the markers PECAM1 and ACTA2, respectively (Figures 1B,C).

### Characterization of myeloid cell subsets

**FIGURE 2:**
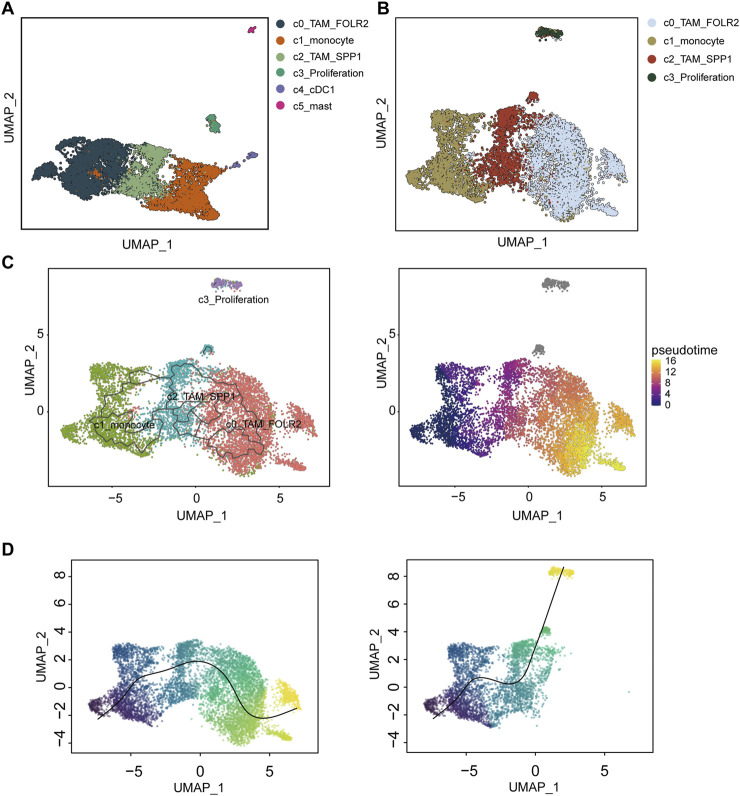
Differentiation and trajectory analysis of tumor-associated macrophages (TAMs) in hepatocellular carcinoma (HCC). **(A)** UMAP clustering of myeloid cells re-clustered into six distinct populations: C0_TAM_FOLR2 (dark blue), C1_monocyte (orange), C2_TAM_SPP1 (green), C3_Proliferation (light green), C4_cDC1 (purple), and C5_mast (pink). **(B)** UMAP visualization of the differentiation trajectories for the TAM-related subpopulations C0_TAM_FOLR2, C1_monocyte, C2_TAM_SPP1, and C3_Proliferation, with distinct clusters identified in the analysis. **(C)** Trajectory analysis using the Monocle3 method, highlighting the differentiation path of macrophages from C1_monocyte, with a transition to C0_TAM_FOLR2 and C3_Proliferation. **(D)** Slingshot-based trajectory analysis showing two differentiation trajectories, with one path linking C1_monocyte to C0_TAM_FOLR2 and the other leading to C3_Proliferation. The color gradient in **(C,D)** represents pseudotime, with warmer colors indicating later stages of differentiation. UMAP, Uniform Manifold Approximation and Projection.

Myeloid cells were re-clustered into six distinct populations (Figure 2A). Cluster C0_TAM was characterized by high expression of FOLR2 and MRC1 (c0_TAM_FOLR2) (Supplementary Figure S1A). Cluster C1 exhibited high expression of FCN1 and S100A8, and was identified as monocytic cells (c1_monocyte) (Supplementary Figure S1B). Cluster C2_TAM showed high expression of SPP1 and FN1 (c2_TAM_SPP1) (Supplementary Figure S1C). Cluster C3, characterized by high expression of MKI67 and TOP2A, was identified as a proliferation-associated macrophage population (c3_Proliferation) (Supplementary Figure S1D). Cluster C4 displayed high expression of XCR1 and CLEC9A, and was identified as dendritic cells (DCs) (c4_cDC1) (Supplementary Figure S1E). Finally, Cluster C5 showed high expression of TPSB2 and TPSAB1, which were identified as mast cells (c5_mast) (Supplementary Figure S1F).

### Differentiation and trajectory analysis of tumor-associated macrophages in hepatocellular carcinoma

To investigate the differentiation of TAMs, four macrophage-related subpopulations-C0_TAM_FOLR2, C1_monocyte, C2_TAM_SPP1, and C3_Proliferation-were extracted and re-clustered for dimensionality reduction (Figure 2B). Both Monocle3 and Slingshot were employed to explore the differentiation trajectories of macrophages (Figures 2C,D). Interestingly, in both Monocle3 and Slingshot, C1_monocyte was identified as the starting point of macrophage differentiation. In Monocle3, some cells in C0_TAM_FOLR2 were considered to represent a later stage of differentiation (Figure 2C). In Slingshot, two distinct differentiation trajectories were observed for macrophages: one starting from C1_monocyte and ultimately differentiating into C0_TAM_FOLR2, and another connecting to C3_Proliferation (Figure 2D).

### Functional characterization and comparison of TAM subpopulations in the tumor microenvironment

**FIGURE 3:**
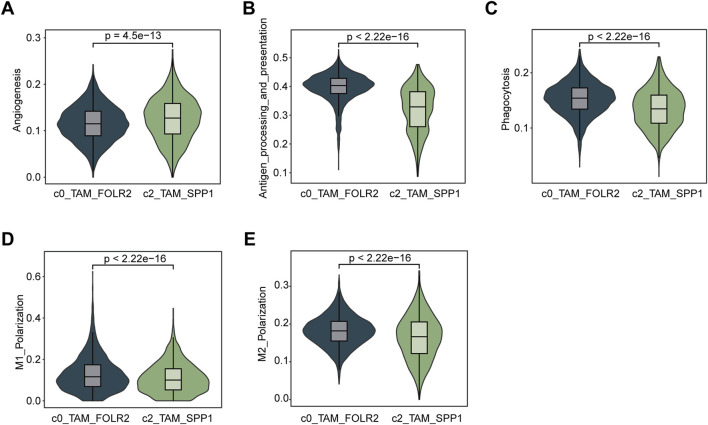
Functional characterization and comparison of C0_TAM_FOLR2 and C2_TAM_SPP1 subpopulations in the tumor microenvironment. **(A)** Violin plot showing the activity of angiogenesis pathways in the C0_TAM_FOLR2 and C2_TAM_SPP1 subpopulations. C2_TAM_SPP1 exhibited significantly higher angiogenesis activity. **(B)** Violin plot depicting antigen processing and presentation (KEGG) scores, with C0_TAM_FOLR2 showing higher activity compared to C2_TAM_SPP1, suggesting a stronger role in antigen processing. **(C)** Violin plot of phagocytosis scores, indicating no significant difference between the two subpopulations, though C0_TAM_FOLR2 showed slightly stronger phagocytic activity. **(D)** Violin plot showing M1 polarization scores, with C0_TAM_FOLR2 displaying higher activity in M1 polarization, reflecting its involvement in pro-inflammatory responses. **(E)** Violin plot for M2 polarization scores, showing a marginally higher score in C0_TAM_FOLR2 compared to C2_TAM_SPP1, although the difference was small. The statistical significance (*p*-values) for all comparisons is shown above each plot.

To examine the functional differences between the C0_TAM_FOLR2 and C2_TAM_SPP1 subpopulations, we utilized AUCell to calculate their scores based on various functional gene sets, including angiogenesis (Figure 3A), antigen processing and presentation (Figure 3B), phagocytosis (Figure 3C), M1 polarization (Figure 3D), and M2 polarization (Figure 3E). The results showed that C2_TAM_SPP1 exhibited higher activity in angiogenesis, indicating a stronger involvement in this process compared to C0_TAM_FOLR2. In contrast, C0_TAM_FOLR2 demonstrated higher antigen processing and presentation capacity, along with a more prominent role in M1 polarization, suggesting its involvement in pro-inflammatory responses. Although the difference in M2 polarization scores was small, C0_TAM_FOLR2 showed slightly higher scores than C2_TAM_SPP1. Furthermore, while both subpopulations exhibited similar levels of phagocytic activity, C0_TAM_FOLR2 showed a marginally stronger phagocytic ability, suggesting a more pronounced role in pathogen clearance.

### Causal associations between SPP1^+^ TAM-Related genes and HCC risk

**FIGURE 4:**
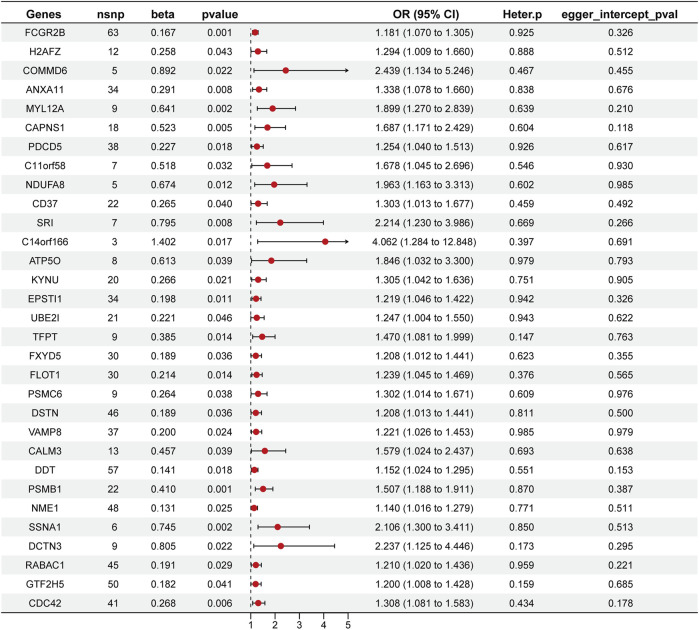
Forest plot of Mendelian randomization analysis identifying SPP1^+^ TAM-related genes associated with overall survival (OS). This plot presents the results of the MR analysis for 31 genes associated with SPP1^+^ TAMs and their impact on OS in HCC. The plot displays the odds ratios (OR) with 95% confidence intervals (CI) for each gene, indicating their respective effects on OS. Red dots represent the point estimate, while the horizontal lines show the 95% CI.

Through a two-sample MR analysis of 832 SPP1^+^ TAM-related genes (Supplementary Table S1), we identified 31 genetically predicted gene expressions showing significant positive associations with HCC risk (IVW method: *β* > 0, *p* < 0.05). These included FCGR2B (*β* = 0.167, OR = 1.181, 95% CI: 1.070–1.305; *p* = 0.001), SRI (*β* = 0.795, OR = 2.214, 95% CI: 1.230–3.986; *p* = 0.008), and NDUFA8 (*β* = 0.674, OR = 1.963, 95% CI: 1.1.163–3.313; *p* = 0.012), among others (complete list in Figure 4).

Assessment of heterogeneity using the IVW Cochran’s Q test revealed no statistically significant heterogeneity (Figure 4). The MR-Egger intercept analysis further demonstrated the absence of horizontal pleiotropy in these genetic associations (Figure 4). Collectively, these methodological validation analyses confirm the robustness and reliability of our MR findings.

### Construction of STRS for HCC patients

**FIGURE 5:**
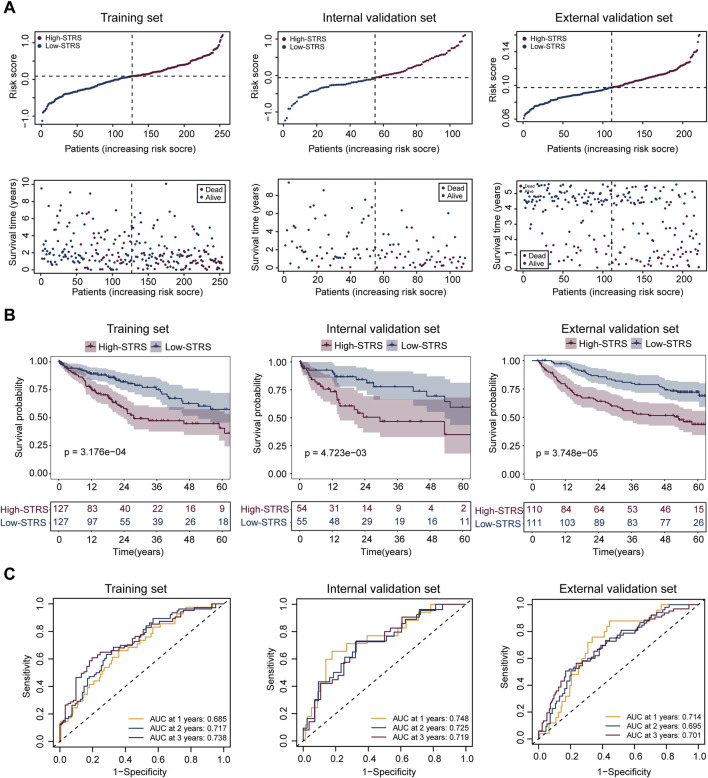
Prognostic performance of the risk score model in HCC patients across multiple cohorts. **(A)** Risk score distribution and survival status of patients in the training set, internal validation set, and external validation set. Patients were divided into high- and low-STRS groups based on the median risk score in each cohort. Survival time is shown for each patient, with blue representing alive patients and red representing deceased patients. **(B)** Kaplan-Meier survival curves for OS in the high- and low-STRS groups in the training set, internal validation set, and external validation set. **(C)** Receiver operating characteristic (ROC) curve analysis for 1-year, 2-year, and 3-year OS in the training set, internal validation set, and external validation set.

In the training set, the 31 genes selected through MR were used for LASSO regression to identify the most predictive genes as OS-related indicators (Supplementary Figure S2). Ultimately, 16 SPP1^+^TAM-related genes associated with OS were identified (FCGR2B, CAPNS1, C11orf58, NDUFA8, CD37, SRI, KYNU, UBE2I, TFPT, PSMC6, CALM3, DDT, NME1, RABAC1, GTF2H5, CDC42) (Supplementary Table S2). In the analysis of risk scores, patients were divided into high- and low-STRS groups based on the median risk score in each cohort: training set, internal validation set, and external validation set (Figure 5A). For each cohort, a clear separation in survival time was observed between the high- and low-STRS groups. Specifically, patients in the high-STRS group exhibited significantly worse survival outcomes compared to those in the low-STRS group (Figure 5A). Kaplan-Meier survival analysis further demonstrated a marked difference in OS between high- and low-STRS patients in all three cohorts. In the training set, the survival probabilities were significantly lower in the high-STRS group (*p* = 3.176e-04), and similar trends were observed in both the internal validation set (*p* = 4.723e-03) and the external validation set (*p* = 3.748e-05), indicating that the risk score is a robust prognostic factor across different datasets (Figure 5B). Receiver operating characteristic (ROC) curve analysis was performed to assess the predictive performance of the risk score for 1-year, 2-year, and 3-year OS. The AUC values for the training set were 0.685, 0.717, and 0.738 for 1-, 2-, and 3-year OS, respectively, demonstrating a strong predictive ability. For the internal validation set, AUC values were 0.748, 0.725, and 0.719, and for the external validation set, AUC values were 0.714, 0.739, and 0.730 for 1-, 2-, and 3-year OS, respectively (Figure 5C).

**FIGURE 6:**
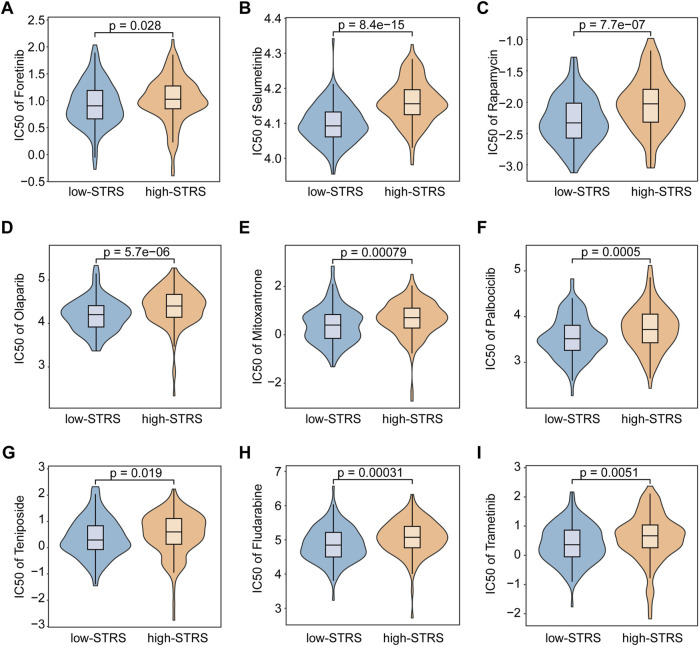
Drug sensitivity analysis. Comparative analysis of *Foretinib* **(A)**, *Selumetinib* **(B)**, *Rapamycin* **(C)**, *Olaparib* **(D)**, *Mitoxantrone* **(E)**, *Palbociclib* **(F)**, *Teniposide* **(G)**, *Fludarabine* **(H)**, and *Trametinib* **(I)** efficacy in high- versus low-STRS groups.

Drug sensitivity analysis revealed a significant difference in drug sensitivity between the high- and low-STRS groups. The high-STRS group exhibited higher IC50 values for multiple drugs (Figure 6). These findings indicate that the risk score model has high accuracy in predicting patient survival across multiple cohorts. Furthermore, the model is also able to effectively predict drug sensitivity, providing strong support for personalized treatment.

### Oncogenic role of the prognostic marker gene UBE2I in HCC

**FIGURE 7:**
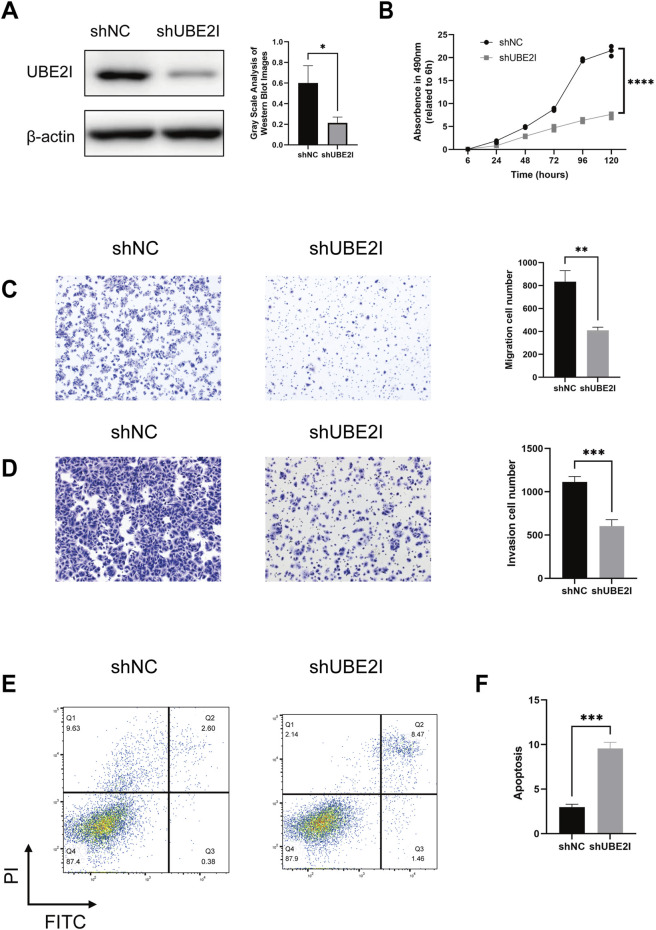
UBE2I depletion impairs the proliferation, migration and survival of HCC cells. **(A)** Western blot confirmation of UBE2I depletion using two independent short hairpin RNA (shRNA) constructs in Huh7 cells. Left panels show representative images, while the right panels present quantification data. **(B)** Cell viability assessment via the Cell Counting Kit-8 (CCK-8) assay in UBE2I-depleted and control Huh7 cells. **(C,D)** Transwell migration and invasion assays comparing UBE2I-depleted and control Huh7 cells. Left panels show representative images, while the right panels present quantification data. **(E,F)** Annexin V/PI flow cytometry analysis of UBE2I-depleted and control Huh7 cells. Left panels display representative images, and the right panels show quantification data. Data are presented as mean ± SD. *, *p* < 0.05, **; *p* < 0.01***, *p* < 0.001 (one-way ANOVA; Student’s t-test). shNC, negative control shRNA. All *in vitro* experiments were repeated biologically three times.

To gain further insight into the underlying mechanisms of the STRS, we investigated the genes associated with this model, particularly focusing on UBE2I, which has been less extensively studied. Kaplan-Meier survival analysis of UBE2I expression was conducted in the training set, internal validation set, and external validation set. The training set (*p* = 3.837e-02) showed significant associations between higher UBE2I expression and poorer survival, while the internal validation set (*p* = 8.287e-02) and external validation set (*p* = 4.675e-01) did not reach statistical significance (Supplementary Figure S3A). We then explored the function of UBE2I in the progression of HCC. Using shRNA, we knocked down UBE2I expression in the HCC cell line Huh7 (Figure 7A; Supplementary Figure S3B). Our results showed that the knockdown of UBE2I inhibited cell growth in the Huh7 cell line (Figure 7B) and significantly impaired cell migration and invasion abilities (Figures 7C,D; Supplementary Figure S3C). Additionally, UBE2I knockdown notably induced apoptosis in Huh7 cells (Figures 7E,F; Supplementary Figure S3D).

## Discussion

Our study provides significant contributions to the understanding of HCC by identifying distinct TAMs subpopulations and their functional roles within the tumor microenvironment. Through a combination of single-cell transcriptomics and MR analysis, we identified key genes associated with HCC progression, enabling the construction of a robust prognostic model. The model, based on 16 SPP1^+^ TAMs-related genes, offers a novel and powerful tool for stratifying HCC patients, which may improve individualized treatment strategies. By bridging the gap between single-cell analysis and causal genetic findings, our study paves the way for future clinical applications aimed at improving patient outcomes and guiding therapeutic decisions in HCC.

Similar to previous studies, we further classified macrophages, with TAMs being subdivided into four categories. Interestingly, different pseudotime analyses exhibited similar trends. Both Monocle3 and Slingshot regarded C1_monocyte as the starting point of macrophage differentiation, suggesting that the differentiation from monocytes to TAMs is a crucial immunological process within the tumor microenvironment. In addition to the differentiation trajectory from monocle to mature TAMs, Slingshot analysis identified another distinct trajectory, in which cells differentiate from monocle to mature TAMs and subsequently transition into proliferation-associated macrophages. This phenomenon may be closely related to the self-renewal capacity of macrophages. Recent studies have demonstrated that mature macrophages are capable of self-renewal through local proliferation, a process that is particularly important during inflammatory responses, as it enables a rapid expansion of macrophage populations independent of continuous input from circulating monocytes (Sieweke and Allen, 2013).

The C0_TAM_FOLR2 subset showed high expression of FOLR2 and MRC1. Multiple studies have demonstrated that FOLR2^+^ TAMs play a key role in tumor initiation and progression, especially in regulating immune responses. A study in breast cancer found that FOLR2^+^ TAMs localized around the blood vessels in the tumor stroma, where they interact with CD8^+^ T cells. Further *in vitro* experiments confirmed that FOLR2^+^ TAMs could effectively activate effector CD8^+^ T cells (Nalio et al., 2022). In contrast, in HCC, FOLR2^+^ macrophages exhibited a fetal-like reprogramming that enhanced the immune-suppressive state in tumor tissues, similar to the immune characteristics observed during liver development (Sharma et al., 2020). In our study, however, FOLR2^+^ TAMs exhibited stronger antigen-presenting and phagocytic abilities. These differences highlight the complexity of the functional roles of FOLR2^+^ macrophages in different tumor microenvironments. Further research is crucial for elucidating their mechanistic roles in various types of tumors and their potential clinical significance. The identification of C2_TAM_SPP1 is consistent with recent studies, which have all indicated its negative impact on patient prognosis. Zhang et al. (2020) reported that TAM-SPP1^+^ is associated with angiogenesis in colorectal cancer, while in our functional analysis, C2_TAM_SPP1 showed a higher angiogenesis score. Tumor angiogenesis remains another critical aspect of HCC progression. A recent study using single-cell spatial transcriptomics emphasized the importance of angiogenesis in HCC treatment (Ye et al., 2024). The importance of immune modulation in HCC progression is equally critical. Recent studies have shown that SPP1^+^ TAMs play a critical role in immune modulation within the TME (Wang B. et al., 2024). A study in HCC demonstrated that the interaction between SPP1^+^ macrophages and POSTN^+^ cancer-associated fibroblasts (CAFs) increased SPP1 expression through the IL-6/STAT3 signaling pathway, forming an immune-suppressive microenvironment and limiting the effectiveness of immunotherapy (Wang H. et al., 2024). Another study demonstrated that the tumor immune barrier (TIB), formed by the interaction between SPP1^+^ TAMs and CAFs, restricts immune cell infiltration into the tumor core, thereby reducing the efficacy of immunotherapy. The study showed that blocking SPP1 or deleting Spp1 specifically in macrophages disrupted this immune barrier, enhancing T-cell infiltration and improving the response to anti-PD-1 treatment. This suggests that targeting SPP1^+^ TAMs could be an effective strategy to enhance immunotherapy efficacy in HCC (Liu et al., 2023). Another study identified the key role of SPP1^+^ TAMs in AFP-positive hepatocellular carcinoma (APHC), which is associated with a suppressive tumor microenvironment. The study found that SPP1^+^ TAMs was enriched in APHC, along with elevated CD44 expression on both T cells and tumor cells. Targeting the SPP1-CD44 axis restored T cell function and reduced tumor burden in mouse models, suggesting that this axis could be a promising therapeutic target to enhance immune responses in APHC (He et al., 2023). Furthermore, we found that the traditional “classically activated” (M1) and “alternatively activated” (M2) classification cannot effectively represent the functions of different macrophage subsets at the single-cell level, a finding consistent with previous studies (Zhang et al., 2020; Azizi et al., 2018).

In this study, we were the first to investigate the causal relationship between SPP1^+^ TAM-related genes and cancer using MR. With the advancement of genomics, increasing evidence has revealed the significant role of genetics in the etiology of diseases. MR, as a powerful causal inference method, utilizes genetic variants that are closely associated with disease exposure and are not affected by confounding factors, enabling an effective identification of causal relationships between gene expression and disease (Brennan et al., 2011). Our findings show that the expression of 31 genetically predicted genes is significantly positively correlated with HCC risk, further validating the potential role of these genes in hepatocellular carcinoma. For instance, the FCGR2B gene, which encodes the FcγRIIB receptor, was identified as one of the genes significantly associated with HCC risk in our study (β = 0.167, OR = 1.181, *p* = 0.001). This finding is consistent with previous studies, which have indicated that FCGR2B plays an important role in the tumor immune microenvironment, particularly in regulating immune cell activity and influencing tumor immune evasion (Ku et al., 2024). Our MR analysis further confirmed the potential pathogenic role of FCGR2B in liver cancer, revealing its possible mechanism as an immune regulatory factor in the pathogenesis of hepatocellular carcinoma. Furthermore, our MR analysis did not reveal significant heterogeneity, nor did we find evidence of horizontal pleiotropy, which further confirms the robustness and reliability of these genetic associations. These results not only expand our understanding of the relationship between SPP1^+^ TAMs-related genes and HCC but also provide theoretical support for the potential clinical application of these genes as biomarkers in the future.

In our study, we successfully developed a prognostic signature comprising 16 SPP1^+^ TAMs-related genes (FCGR2B, CAPNS1, C11orf58, NDUFA8, CD37, SRI, KYNU, UBE2I, TFPT, PSMC6, CALM3, DDT, NME1, RABAC1, GTF2H5, CDC42), which demonstrated the potential to predict OS in patients with HCC. The robustness of this risk score model was confirmed through its ability to stratify patients into high- and low-STRS groups, with significantly different survival outcomes across multiple cohorts, including the training set, internal validation set, and external validation set. These results highlight the reliability and strong predictive performance of the identified gene signature.

To deepen the understanding of STRS, among the 16 genes identified in our signature, UBE2I was selected for further experimental investigation. UBE2I, a small ubiquitin-like modifier E2 enzyme, has been shown to be highly expressed in various tumors. Consistent with previous studies, our functional validation results demonstrated that the knockdown of UBE2I significantly inhibited the growth, migration, and invasion abilities of HCC tumor cell lines, while simultaneously inducing apoptosis (Wang et al., 2020). These findings highlight the critical role of UBE2I in HCC progression and its potential as a therapeutic target, further supported by its high expression in tumors and strong association with poor prognosis. Similarly, CDC42, a member of the Rho GTPase family, has been reported to facilitate invadopodia formation, promoting metastasis in HCC. It is important to recognize that the remaining 14 genes in the signature also warrant further investigation. Each of these genes may play a complementary role in shaping the tumor microenvironment and influencing overall survival in HCC patients. Therefore, our findings highlight the potential of the 16-gene signature as a comprehensive prognostic tool, offering valuable insights into the molecular landscape of HCC and paving the way for more personalized treatment approaches.

Additionally, drug sensitivity analysis revealed a significant difference in drug sensitivity between the two patient groups. The IC50 of *Docetaxel* and *Epirubicin* in the low-STRS group was significantly lower than that in the high-STRS group, suggesting that for patients who are inoperable, low-STRS patients with STRS may benefit more from transcatheter arterial chemoembolization (TACE). Foretinib is an oral multi-kinase inhibitor targeting MET, ROS, RON, AXL, TIE-2, and VEGFR (Choueiri et al., 2013), which has demonstrated good anti-tumor activity and tolerability in a phase I/II single-arm study (Yau et al., 2017). It is obvious that the STRS helps to identify potential drug-sensitive and drug-resistant patients. This further underscores the importance of incorporating drug sensitivity profiling into the clinical management of HCC, potentially guiding more personalized and effective treatment strategies. Combining this with the prognostic model, our findings could lead to better patient stratification, ensuring that patients receive treatments tailored to their specific tumor profiles and drug sensitivities.

While our study provides valuable insights into the functional differences between TAM subpopulations in HCC, there are certain limitations that need to be addressed. The data used in this analysis were derived from publicly available single-cell transcriptome datasets, which may not fully capture the complexity of tumor-immune interactions across diverse HCC patient populations. Additionally, although we identified key genes through Mendelian randomization, the functional validation of these genes *in vivo* remains an important next step.

The STRS model, based on 16 SPP1^+^ TAMs-related genes, provides a powerful tool for stratifying patients with HCC and could guide individualized treatment strategies. Clinically, the model may be used to identify high-risk patients who require more aggressive treatment and low-risk patients who could benefit from less intensive therapies, thus improving patient outcomes. However, the STRS model does have some limitations. It relies on gene expression profiles, which may vary across different patient populations and tumor subtypes. Moreover, the model does not incorporate clinical factors, such as comorbidities, that could influence patient outcomes. To further improve its clinical applicability, future studies should validate the STRS model in larger, independent cohorts and explore how it interacts with clinical factors to refine risk stratification. In the future, incorporating spatial transcriptomics could provide more insights into the spatial interactions of TAMs within the tumor microenvironment, potentially enhancing the precision of the STRS model. This would allow for a better understanding of TAM function, tumor progression, and therapy resistance, further supporting the potential of STRS in clinical practice.

## Data Availability

The datasets presented in this study can be found in online repositories. The names of the repository/repositories and accession number(s) can be found in the article/Supplementary Material.

## Supplementary material

SUPPLEMENTARY FIGURE S1
Expression of marker genes across different myeloid cell subsets in HCC. **(A)** UMAP plots showing the expression of FOLR2 and MRC1 for cluster C0_TAM_FOLR2, characterized by tumor-associated macrophages (TAMs) with these markers. **(B)** FCN1 and S100A8 expression in cluster C1_monocyte, identified as monocytic cells. **(C)** SPP1 and FN1 expression in cluster C2_TAM_SPP1, identified as SPP1^+^ TAMs. **(D)** MKI67 and TOP2A expression in cluster C3_Proliferation, associated with proliferation-driven macrophage populations. **(E)** XCR1 and CLEC9A expression in cluster C4_cDC1, identifying dendritic cells (DCs). **(F)** TPSB2 and TPSAB1 expression in cluster C5_mast, identified as mast cells. The density plots show gene expression levels, with darker blue indicating higher expression in each subset.

SUPPLEMENTARY FIGURE S2
LASSO regression for identifying predictive genes associated with overall survival (OS) in the training set. **(A)** The partial likelihood deviance plot for the LASSO regression model. The red dotted line indicates the optimal value of the regularization parameter (λ), which is selected based on the minimum deviance. **(B)** Coefficient profiles for each gene in the LASSO regression model. The coefficients are plotted against the log-transformed values of the regularization parameter (λ). The different colored lines represent individual genes, and the shrinking of coefficients with increasing λ is observed.

SUPPLEMENTARY FIGURE S3
Additional supporting data for UBE2I depletion effects in Huh7 cells. **(A)** Kaplan-Meier survival analysis of UBE2I expression in the training set (left), internal validation set (middle), and external validation set (right). The analysis shows the association between UBE2I expression and overall survival across all three cohorts, supporting the prognostic value of UBE2I. **(B)** Full-length Western blot images corresponding to Figure 7A, showing all detected bands for UBE2I and loading controls (β-actin). **(C)** Representative images of remaining replicates from Transwell migration and invasion assays (related to Figures 7C,D). **(D)** Complete flow cytometry scatter plots of Annexin V/PI staining for apoptosis analysis (supporting Figures 7E,F).

SUPPLEMENTARY TABLE S1
List of genes related to SPP1^+^ TAMs.

SUPPLEMENTARY TABLE S2
LASSO-selected 16 SPP1^+^ TAM-related genes and their corresponding coefficients.
