# Supplementary figures and images for: Integrative multi-omics and Mendelian randomization analysis reveal SPP1+ tumor-associated macrophage-driven prognostic signature for hepatocellular carcinoma
Source: Front Mol Biosci. 2025 May 1;12:1594610. doi: 10.3389/fmolb.2025.1594610 (PMC12078150; doi:10.3389/fmolb.2025.1594610)

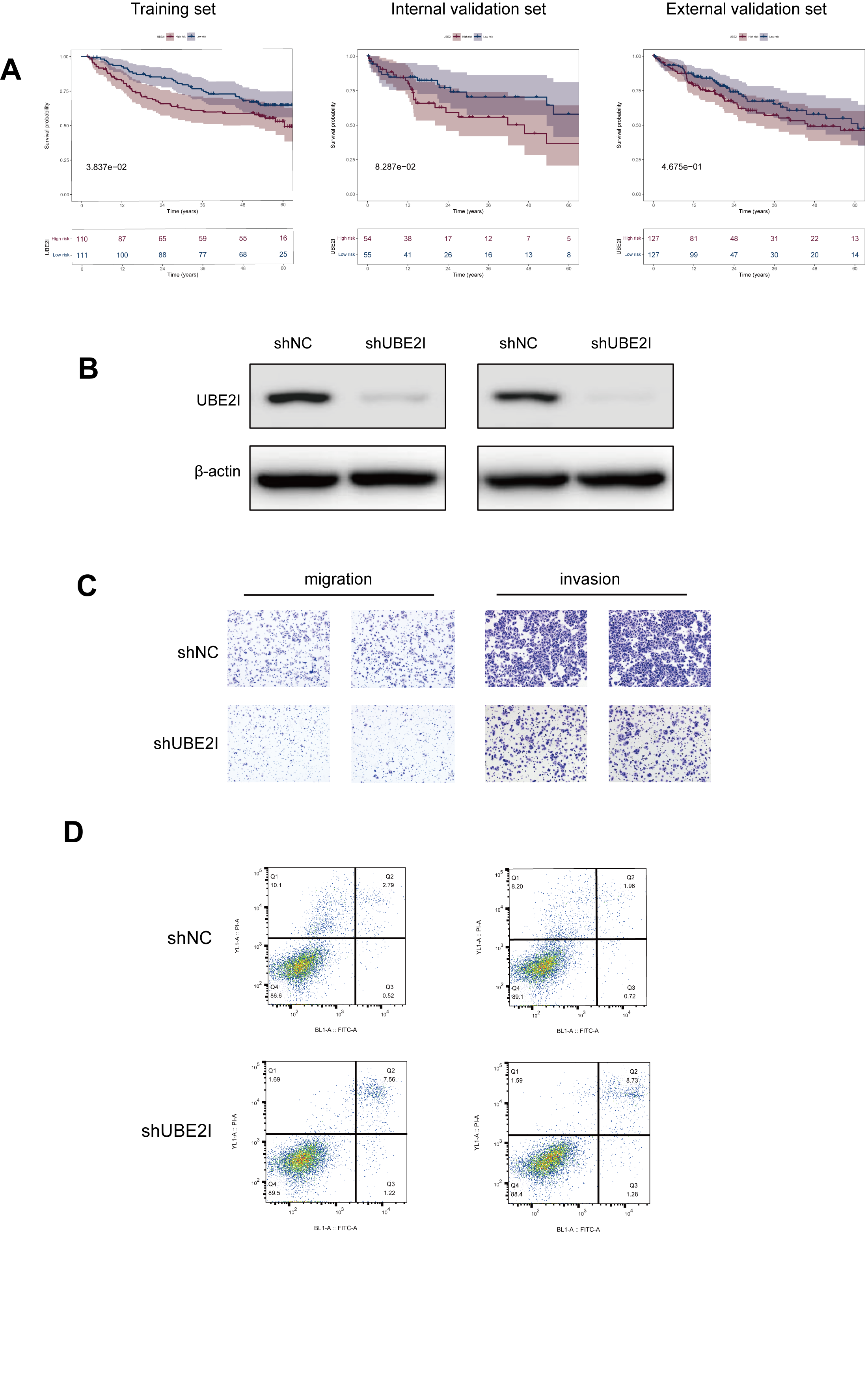

Supplement: Supplementary file 1 [file Image3.tif]

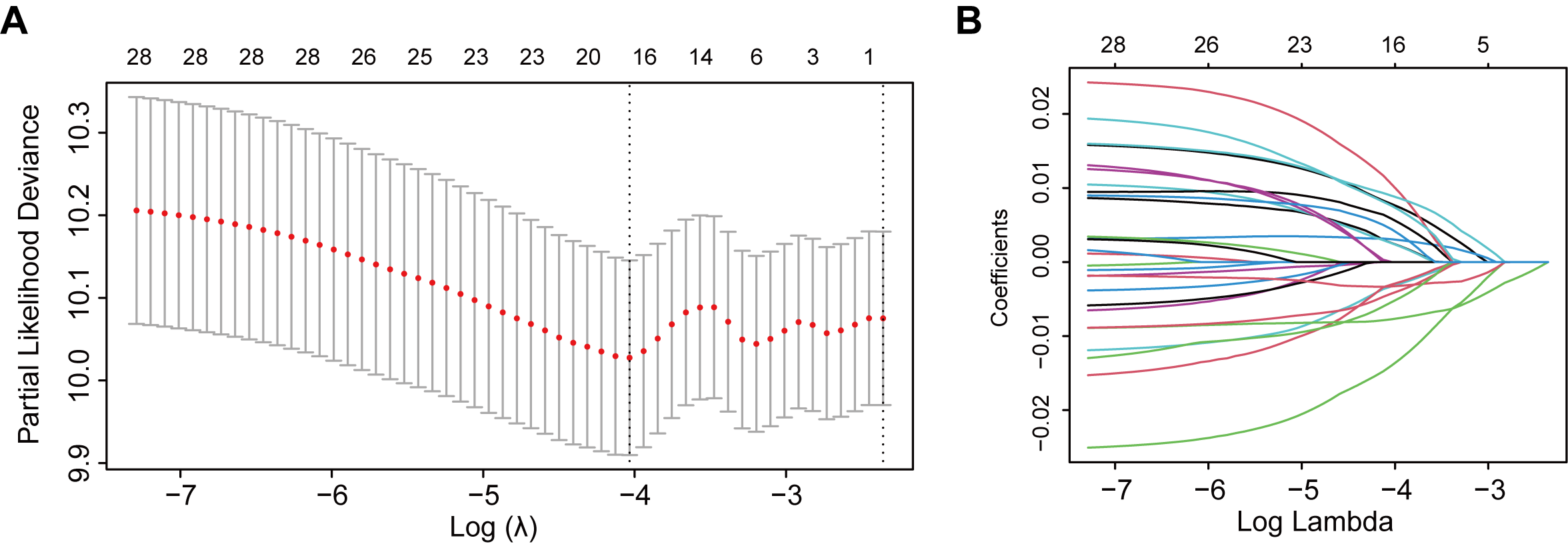

Supplement: Supplementary file 2 [file Image2.tif]

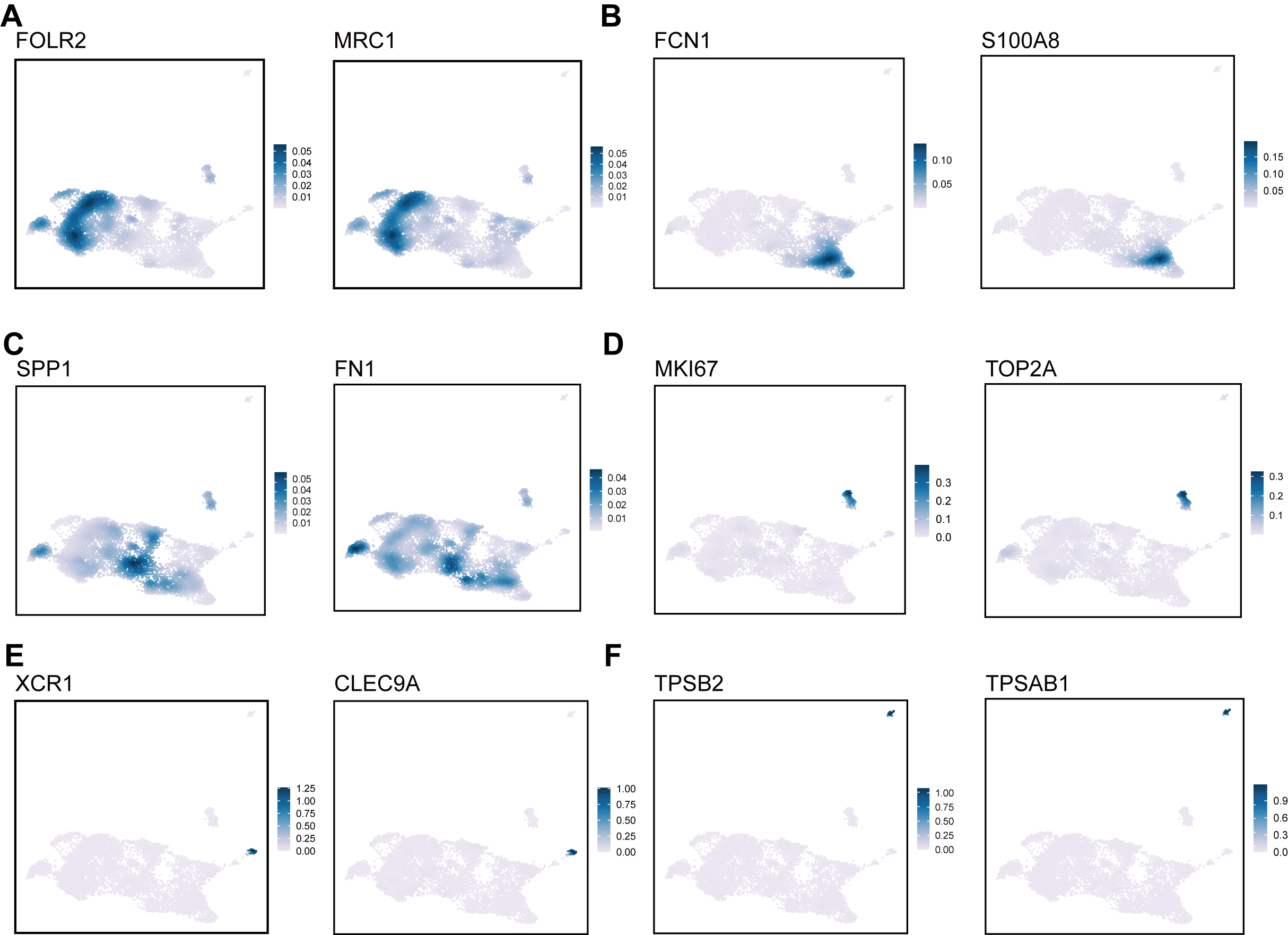

Supplement: Supplementary file 3 [file Image1.tif]
