# Supplementary material for: Integrative multi-omics and Mendelian randomization analysis reveal SPP1+ tumor-associated macrophage-driven prognostic signature for hepatocellular carcinoma
Source: Front Mol Biosci. 2025 May 1;12:1594610. doi: 10.3389/fmolb.2025.1594610 (PMC12078150; doi:10.3389/fmolb.2025.1594610)
Supplement: Supplementary file 4 [file Table2.docx]

**Supplemental Table S2. LASSO-selected 16 SPP1+ TAM-related Genes and Corresponding Coefficients**

| **Genes** | **Coef** |
| --- | --- |
| FCGR2B | 0.00782013438424333  -0.00326971056981184  0.0025486808129732  -0.00302433134035302  -0.0139514861559541  0.00307804788330995  0.00252297191868437  0.00582077522172872  0.0104689358776608  0.00757574578141482  -0.00328950803072879  -0.00772387763353008  0.00894097580412026  -0.00423801676346136  -0.00539329992229897  0.00440326519813866 |
| CAPNS1 |  |
| C11orf58 |  |
| NDUFA8 |  |
| CD37 |  |
| SRI |  |
| KYNU |  |
| UBE2I |  |
| TFPT |  |
| PSMC6 |  |
| CALM3 |  |
| DDT |  |
| NME1 |  |
| RABAC1 |  |
| GTF2H5 |  |
| CDC42 |  |
